# Stevens-Johnson Syndrome: A Perplexing Diagnosis

**DOI:** 10.7759/cureus.7374

**Published:** 2020-03-23

**Authors:** Jonathan Dutt, Amit Sapra, Pooja Sheth-Dutt, Priyanka Bhandari, Supriya Gupta

**Affiliations:** 1 Family and Community Medicine, Southern Illinois University School of Medicine, Springfield, USA; 2 Family Medicine, Southern Illinois University School of Medicine, Springfield, USA

**Keywords:** stevens-johnson syndrome, toxic epidermal necrolysis, skin rash, drug reaction, sepsis, drug rash, fever, skin biopsy, conjunctivitis

## Abstract

Stevens-Johnson syndrome/toxic epidermal necrolysis is a spectrum of mucocutaneous reactions that can occur due to drug reactions, infections with Mycoplasma pneumonia, human immunodeficiency virus (HIV), cancer, and genetics. Stevens-Johnson syndrome involves less than 10% of the body surface, while toxic epidermal necrolysis involves greater than 30%. The most common site of the lesions is mucocutaneous surfaces such as the eyes and oral cavity. Our patient was a 44-year-old female who presented to the emergency department with concerns for pain in her eyes, hands and feet, rash, and sore throat. Her rash worsened during the initial hospitalization. This case emphasizes the importance of pattern recognition of Stevens-Johnson syndrome, as this is a rare but serious condition that must be recognized and treated appropriately. The reaction is most commonly due to medications; however, a thorough history and physical exam are vital to diagnosing this potentially fatal condition.

## Introduction

Stevens-Johnson syndrome and toxic epidermal necrolysis are rare diseases that cause acute destruction of the epithelium of the skin and mucous membrane by a violent immune response [1]. The national incidence per 100,000 was 6.3 for Stevens-Johnson syndrome, 0.7 for Stevens-Johnson syndrome/toxic epidermal necrolysis overlap syndrome, and 0.5 for toxic epidermal necrolysis [2].

Stevens-Johnson syndrome and toxic epidermal necrolysis are known to be life-threatening mucocutaneous reactions, the mortality rates for which are as high as 30%; short and long term morbidities are common. Stevens-Johnson syndrome and toxic epidermal necrolysis are one of the few dermatological diseases that constitute a medical emergency. Early recognition and appropriate and prompt management can be lifesaving [3].

This condition is often misdiagnosed, as in the case of our patient. This can happen due to symptoms that could mimic a plethora of conditions that we commonly see in primary care, such as upper respiratory tract infection, adverse drug reaction, conjunctivitis, viral exanthem, etc. Furthermore, it can be confused with rarer medical conditions such as bullous pemphigoid, and autoimmune blistering diseases (e.g., pemphigus vulgaris, IgA dermatosis) [4].

## Case presentation

We present a case of a 46-year-old African American female with a past medical history of hypertension who presented to the emergency department with concerns for pain in her eyes, hands and feet, rash, and sore throat. She reported that her symptoms started almost a week ago and were gradually getting worse. She worked at a nursing home and reported that half of the rooms were quarantined due to influenza. One week prior to presentation, she noticed she began having pain in her eyes and thought maybe she had gotten sick from one of the residents or her allergies were being triggered. She went home and took allergy medication, and went to bed. She then woke up the next morning and felt significantly worse. Her eyes were very red and were draining along with some blurring in her vision; she also reported her lips were swollen, cracked, red, and had a sore throat.

She reported having had two prior emergency department (ED) visits, the first one being for upper respiratory infection (URI) three weeks ago, for which she was treated with amoxycillin. The second visit three days ago was for similar complaints, during which she was found to be negative for strep throat and influenza and was discharged with an antibiotic ointment for her bilateral conjunctivitis. Her symptoms worsened over the next few days, and she began having swelling and pain in both her palms and soles of her feet. The patient's symptoms continued to worsen for the next few days, so she returned to our emergency room. In the ED, she was in significant pain and wrapped in blankets due to chills. She reported associated rhinorrhea, diarrhea, and an "itchy rash" on her back. Initial lab work was significant for an erythrocyte sedimentation rate (ESR) of 106 and a C-reactive protein (CRP) of 207.9. Her white blood cell count was elevated at 15.0, and her kidney function and liver enzymes were normal. She was given a 1 L intravenous (IV) fluid bolus and tramadol in the ED. She was also found to be negative for influenza and was admitted by our teaching service for further workup and management of her symptoms. During her hospital course, her rash appeared to worsen.

**Figure 1:**
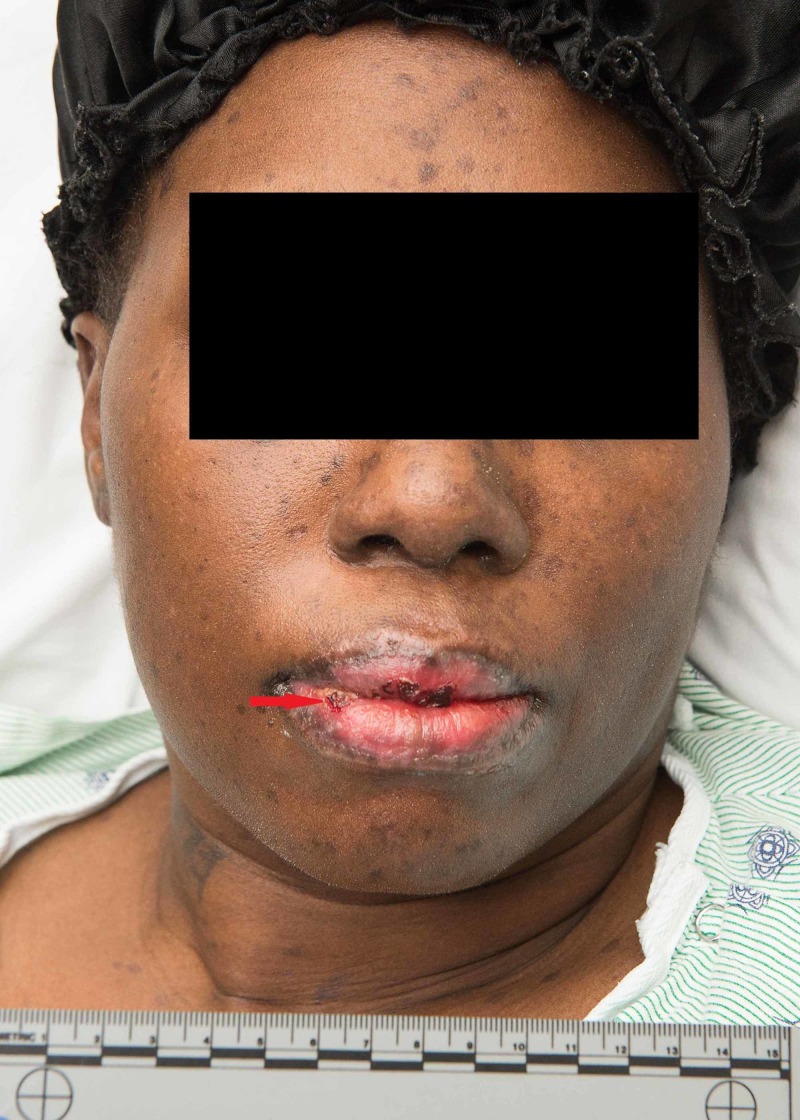
Red arrow shows diffusely eroded lips

The rheumatology team was consulted initially and did an extensive workup, which consisted of human immunodeficiency virus (HIV) screening, antinuclear antibody (ANA), rapid plasma reagin (RPR), flu swab, anti-smith antibodies, anti-cardiolipin antibodies, monospot, SS-A and SS-B antibodies, and antineutrophil cytoplasmic antibodies (ANCA) antibodies, all of which was found to be negative. The dermatology team was also then consulted to evaluate the overwhelming skin findings. The patient had developed diffusely eroded lips and brightly erythematous oral mucosa (Figure 1).

**Figure 2:**
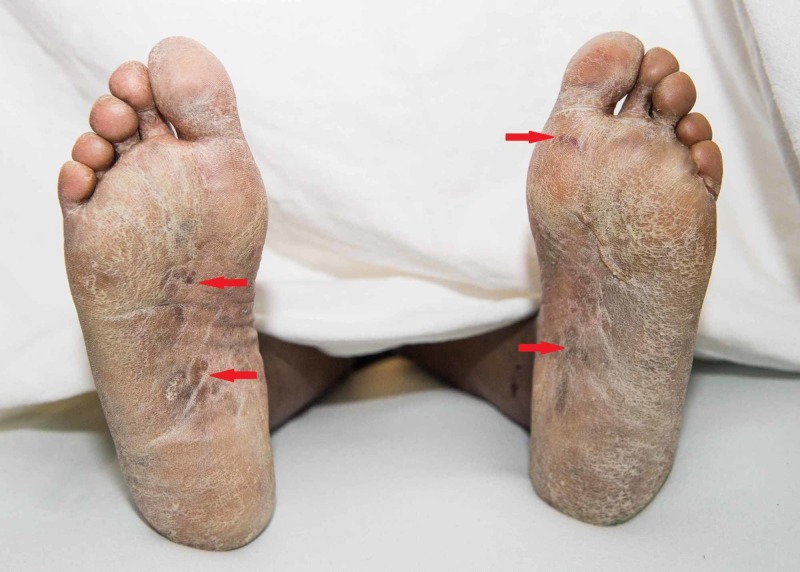
Red arrows show erythematous macules scattered nearly confluently over the soles

Her eyelids were swollen and had irregular purpuric thin papules of varying sizes scattered over her legs, back, chest, and a few on the arms were noted. Some of the papules had peeled to reveal an erythematous base. Erythematous macules were scattered nearly confluently over the palms and soles (Figure 2).

**Figure 3:**
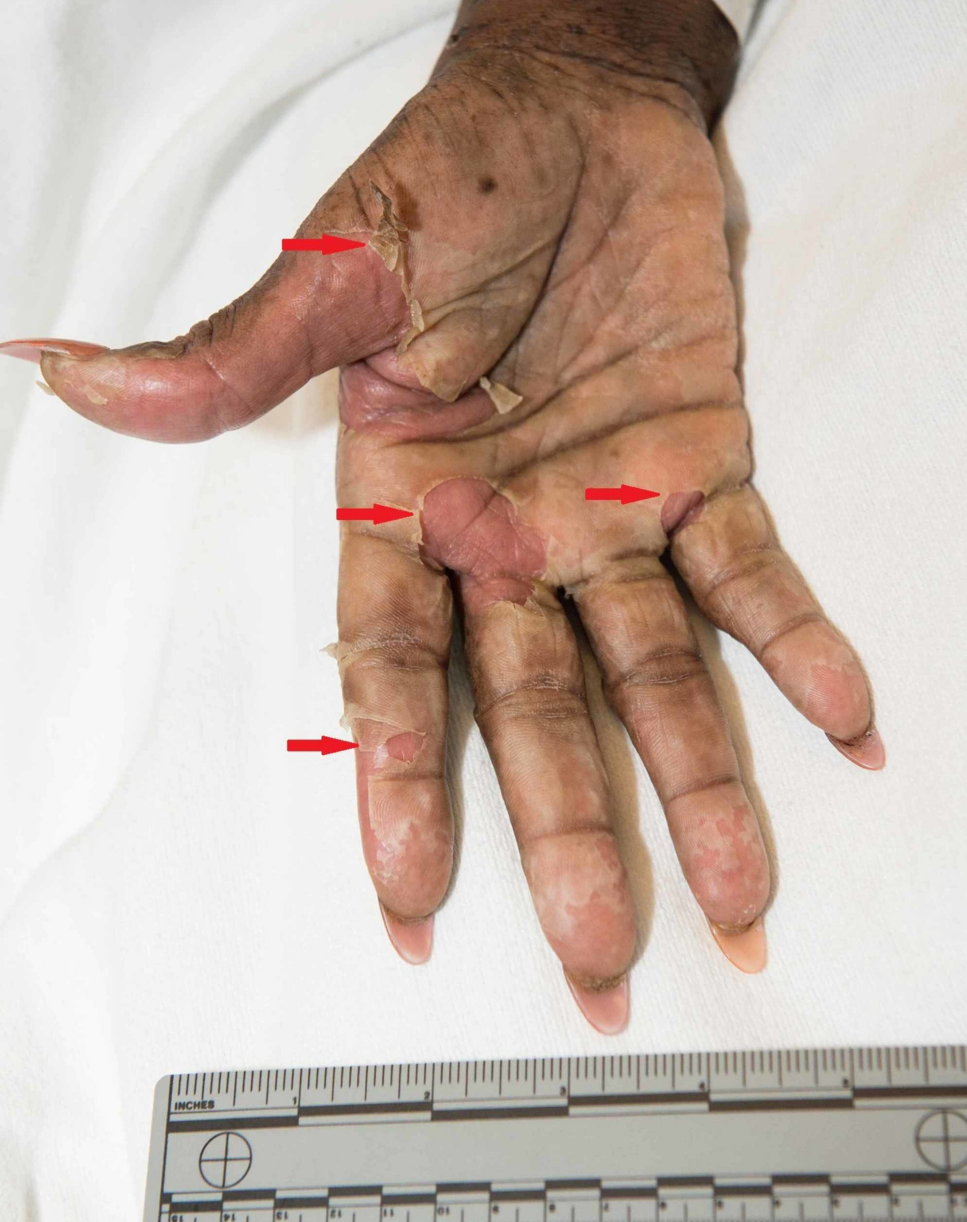
Red arrows point towards the bullae seen over the palmar surface of the hand

**Figure 4:**
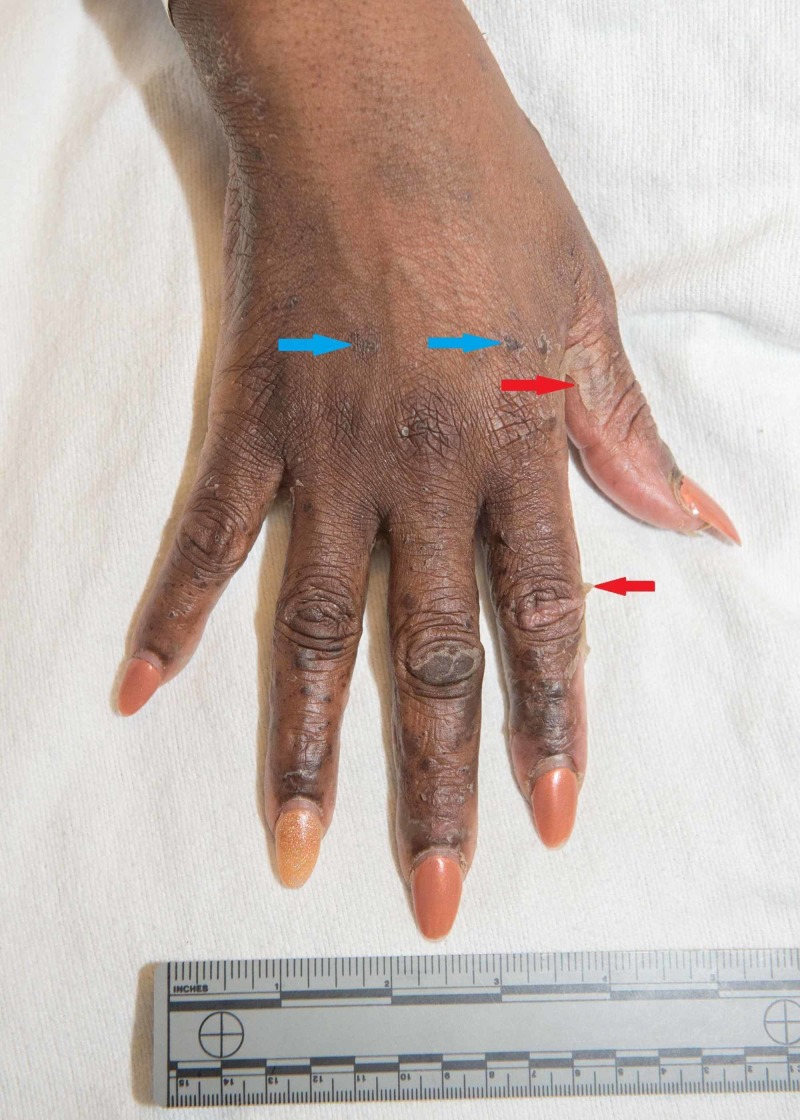
Blue arrows show erythematous macules scattered nearly confluently over the palms and soles; red arrows show bullae

The palms and soles had multiple large deep intact bullae and near confluent erythema covering much of the plantar surface of the toes and distal sole of the foot (Figures 3-4).

**Figure 5:**
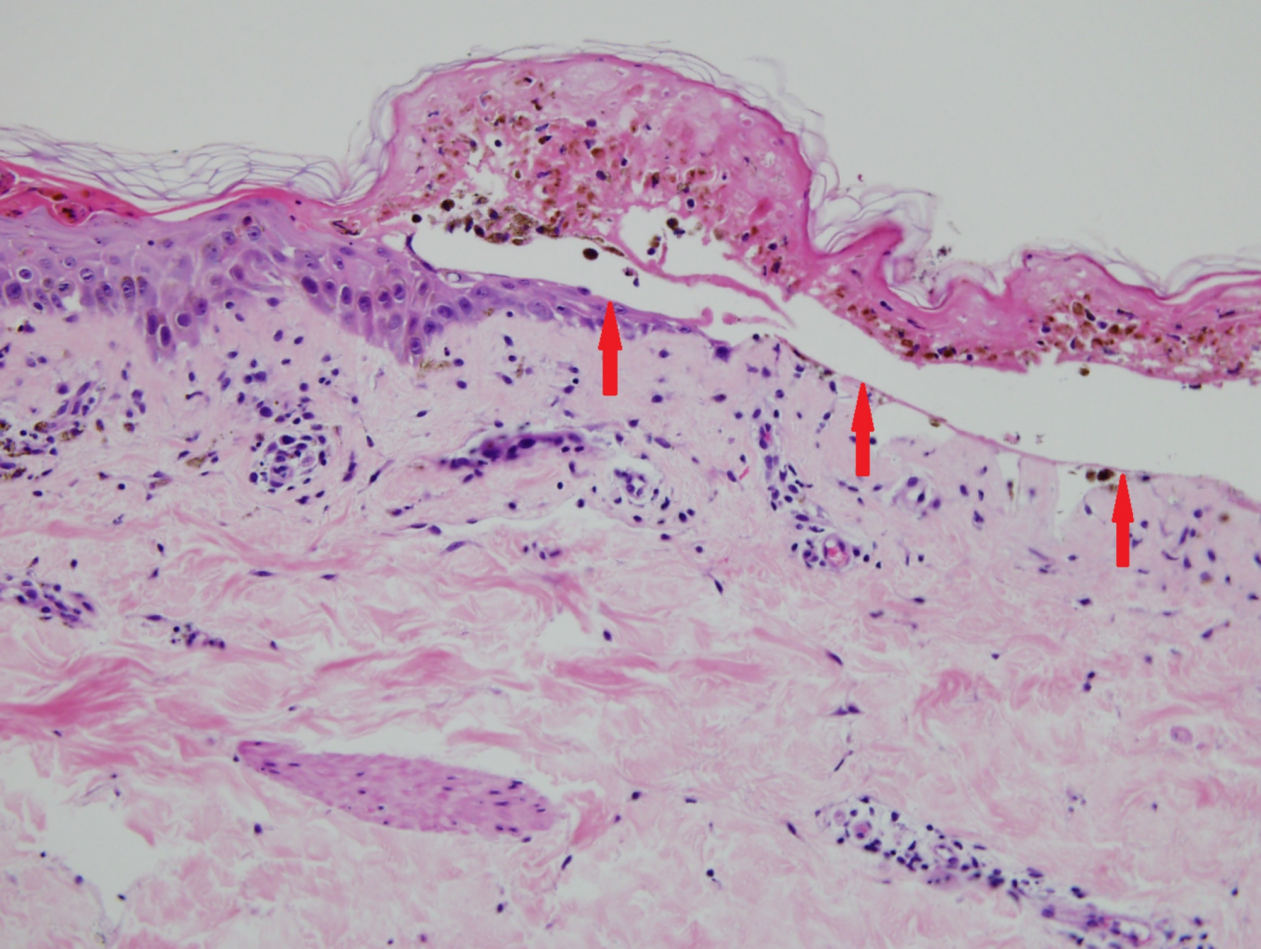
Red arrows showing full-thickness epidermal necrosis and subepidermal bullae

A skin biopsy was performed, which showed full-thickness epidermal necrosis and subepidermal bullae formation (Figure 5).

She also began to spike fevers, and her lips began bleeding and had a purulent discharge. The infectious disease team was consulted, and aztreonam was started, and clindamycin was subsequently added. She continued to spike fevers. Ophthalmology was also consulted for blurry vision and increased discharge. They recommended warm compresses and tobramycin eye drops. Due to continued fevers, an increase in lip bleeding, and concern for infection, the patient was started on vancomycin and acyclovir by the infectious disease team for possible viral infection in the setting of multiple oral mucosal lesions. Viral cultures, acid-fast bacilli culture, fungal culture were all negative, and urine was positive for Staphylococcus saprophyticus.

In the interim, the skin biopsy results were available, and they were found to be consistent with Steven-Johnsons syndrome vs. toxic epidermal necrolysis. The infectious disease team deescalated antibiotic therapy, and her IV antibiotics were stopped.

This case shows the importance of pattern recognition of Steven Johnson's syndrome, as this is a rare but serious condition that must be recognized and treated appropriately.

## Discussion

Stevens-Johnson syndrome and toxic epidermal necrolysis (Lyell disease) are rare diseases that cause acute destruction of the epithelium of the skin and mucous membrane by a violent immune response. Stevens-Johnson syndrome, toxic epidermal necrolysis, and Stevens-Johnson syndrome-toxic epidermal necrolysis overlap are considered variants in a spectrum of disease severity. Stevens-Johnson syndrome involves epidermal detachment of less than 10% of total body surface area, toxic epidermal necrolysis involves epidermal detachment of more than 30% of total body surface area, and Stevens-Johnson syndrome-toxic epidermal necrolysis overlap involves epidermal detachment of 10% to 29% of total body surface area [1].

Fever is often the first symptom. A sore throat, cough, red eyes, and tender, pink skin are early symptoms. A red rash appears, and some areas blister. Blisters are seen in the mouth and on the lips break, leaving sores that make it painful to eat, drink, and swallow. The eyes often feel scratchy, gritty, and dry [5].

A prodrome consisting of fever, arthralgia, and nonspecific upper or lower respiratory symptoms precedes the sudden onset of erythematous blistering of the mucosa and skin in Stevens-Johnson syndrome and toxic epidermal necrolysis. Ocular, oral, and genital mucosal ulcerations appear first and are quickly followed by a generalized painful vesiculobullous rash with extensive necrolysis developing gradually over 2-15 days. Membranes or pseudomembranes may be formed by the mucosal surfaces alongside ulcerative lesions or bullae. The patient may experience recurrent sloughing of epithelial tissues over 4-6 weeks with cycles of lesions lasting around two weeks following re-epithelialization [6].

The differential diagnosis of Stevens-Johnson syndrome/toxic epidermal necrolysis ranges from conditions that we commonly see in primary care, such as upper respiratory tract infection, adverse drug reaction, conjunctivitis, viral exanthem, to rarer medical conditions such as bullous pemphigoid and autoimmune blistering diseases (e.g., pemphigus vulgaris, IgA dermatosis). A definitive diagnosis of Stevens-Johnson syndrome/toxic epidermal necrolysis is made by skin biopsy showing full-thickness dermal necrosis in the absence of immunoglobulin deposition [4].

Management of the acute inflammatory disease consists principally of aggressive and early supportive care effectively done in a burn ICU, much like that of extensive thermal burns. Management of dehydration and proper wound care are critical, as well as monitoring carefully for signs of superinfection that are all too common. Morbidity and mortality are decreased the earlier the offending drug is identified and discontinued. Multiple systemic therapeutic interventions have been proposed to treat Stevens-Johnson syndrome/toxic epidermal necrolysis. Still, all are controversial, and none have proved to help during the course of the disease definitively. Reports are conflicting regarding the efficacy and safety of these treatments, including the most commonly utilized and studied agents, corticosteroids, and IVIG. Short courses of high-dose corticosteroids given in the early stages have been suggested to limit morbidity from the treatment [7]. They have shown potential to hasten recovery, but serious complications have not yet been avoided in these cases. 

A majority of patients recover without major sequelae from the disease. However, even despite prompt management with close observation, Stevens-Johnson syndrome/toxic epidermal necrolysis does pose a significant risk of mortality that is correlated with the extent of skin desquamation, as high as 10% in Stevens-Johnson syndrome, 30% in Stevens-Johnson syndrome/toxic epidermal necrolysis overlap, and almost 50% in toxic epidermal necrolysis [6].

Consultation with ophthalmology, urology, or gynecology departments is necessary to assess organ damage and prevent sequelae [8]. Prognosis is linked to the rapid discontinuation of the offending drug, and it does not seem to be affected by the type or the dose of the causative drug [9].

Our case is a 46-year-old female who was evaluated in the ED twice before she got admitted to the hospital with Stevens-Johnson syndrome. She was prescribed amoxicillin on the initial visit. Subsequently, she was seen and diagnosed with conjunctivitis and given antibacterial eye drops, which did not improve her symptoms. She was eventually admitted to our hospital service and was evaluated by many specialists leading to the diagnosis of Stevens-Johnson Syndrome.

This case shows how Stevens-Johnson Syndrome can present with symptoms that may camouflage with many pathologies we encounter in our daily practice. This case highlights the importance of considering Stevens-Johnson syndrome/toxic epidermal necrolysis as a differential diagnosis because this is a potentially fatal disease. We must remember this syndrome when evaluating patients with presumed drug allergies, strep throat, or infections with viral etiology.

## Conclusions

Our patient is a 46-year-old African American female who was evaluated in the ED on three occasions for upper respiratory tract infection symptoms, pain and redness in her eyes, pain in the hands and feet, rash, and sore throat. Cap amoxicillin was prescribed to her in the first ED visit. During the second visit, she was diagnosed with conjunctivitis and given antibacterial eye drops, which did not improve her symptoms, ultimately getting admitted at the third ED visit. During this hospitalization, she was evaluated by many specialists leading to the diagnosis of Stevens-Johnson Syndrome. This case shows the importance of pattern recognition in Steven Johnson's syndrome which must be recognized and treated appropriately as it is a rare but serious condition. The reaction is most commonly due to medications; however, a thorough history and physical exam is vital in diagnosing this potentially fatal condition.
